# Neurogenic Dysphagia in Subdural Hematoma

**DOI:** 10.3389/fneur.2021.701378

**Published:** 2022-01-26

**Authors:** Sae-Yeon Won, Simon Krieger, Daniel Dubinski, Florian Gessler, Bedjan Behmanesh, Thomas M. Freiman, Juergen Konczalla, Volker Seifert, Sriramya Lapa

**Affiliations:** ^1^Department of Neurosurgery, University Hospital, Goethe University, Frankfurt, Germany; ^2^Department of Neurology, University Hospital, Goethe University, Frankfurt, Germany

**Keywords:** subdural hematoma, dysphagia, speech and language pathologist, predictor, functional outcome

## Abstract

**Introduction:**

Dysphagia is a common and severe symptom of traumatic brain injury (TBI) affecting up to 78% of patients. It is associated with pneumonia, increased morbidity, and mortality. Although subdural hematoma (SDH) accounts for over 50% of TBI, the occurrence of dysphagia in this subtype has not been investigated yet.

**Methods:**

All patients with SDH admitted to the author's institution between the years 2007 and 2020 were included in the study. Patients with SDH and clinical suspicion for dysphagia received a clinical swallowing assessment by a speech and language pathologist (SLP). Furthermore, the severity of dysphagia was rated according to swallowing disorder scale. Functional outcome was evaluated by the Glasgow outcome scale (GOS).

**Results:**

Out of 545 patients with SDH, 71 patients had dysphagia (13%). The prevalence of dysphagia was significantly lower in the surgical arm compared to the conservative arm (11.8 vs. 21.8%; OR 0.23; *p* = 0.02). Independent predictors for dysphagia were GCS <13 at admission (OR 4.17; *p* < 0.001), cardiovascular disease (OR 2.29; *p* = 0.002), and pneumonia (OR 2.88; *p* = 0.002), whereas the operation was a protective factor (OR 0.2; *p* < 0.001). In a subgroup analysis, right-sided SDH was an additional predictor for dysphagia (OR 2.7; *p* < 0.001). Overall, patients with dysphagia improved significantly under the SLP treatment from the initial diagnosis to hospital discharge (*p* < 0.01). However, a subgroup of patients with the most severe grade of dysphagia showed no significant improvement. Patients with dysphagia had significantly worse outcomes (GOS 1–3) compared to those without dysphagia (48.8 vs. 26.4%; *p* < 0.001).

**Conclusion:**

Dysphagia is a frequent symptom in SDH, and the early identification of dysphagia is crucial regarding the initiation of treatment and functional outcome. Surgery is effective in preventing dysphagia and should be considered in high-risked patients.

## Introduction

Dysphagia is a common symptom in traumatic brain injury (TBI), with a prevalence varying between 37 and 78% (1, 2). It is associated with pneumonia, malnutrition, dehydration, and increased morbidity and mortality (3). It has been shown that early detection of dysphagia allows for immediate intervention, and thereby reduces morbidity, duration of hospitalization, and overall healthcare costs (4). The occurrence of dysphagia depends on the origin and type of TBI (5). In this context, several studies have demonstrated that Glasgow Coma Scale score, severity of brain injury, prolonged use of mechanical ventilation, and tracheostomy are associated with dysphagia incidence, severity, and recovery (1, 6, 7). So far, most of the available studies included all types of brain injuries but lacked a dedicated analysis of the association between the type of injury and the occurrence of dysphagia (1). Hence, data on dysphagia following subdural hematoma (SDH), with an overall incidence of 1.7–20.7 per 100,000 persons per year, is very limited (8). Commonly observed symptoms of SDH include headache, vomiting/nausea, impaired consciousness, hemiparesis, gait impairment, speech disorders, seizure, and urinary incontinence. However, impairment of swallowing function is hardly described, potentially leading to an underestimation of dysphagia in the respective cohort.

Thus, the aims of the study were to (1) investigate the overall incidence of dysphagia in patients with SDH, (2) identify predictors associated with dysphagia, and (3) evaluate the natural course of dysphagia receiving dysphagia treatment by speech-language-pathologist (SLP) undergoing surgical or conservative therapeutic regime.

## Methods

### Ethical Approval

This study was approved by the local institutional review board of the Goethe University Frankfurt (EK 20-826). Written informed consent was waived due to the retrospective character of the study.

### Patients and Data Collection

**Figure 1:**
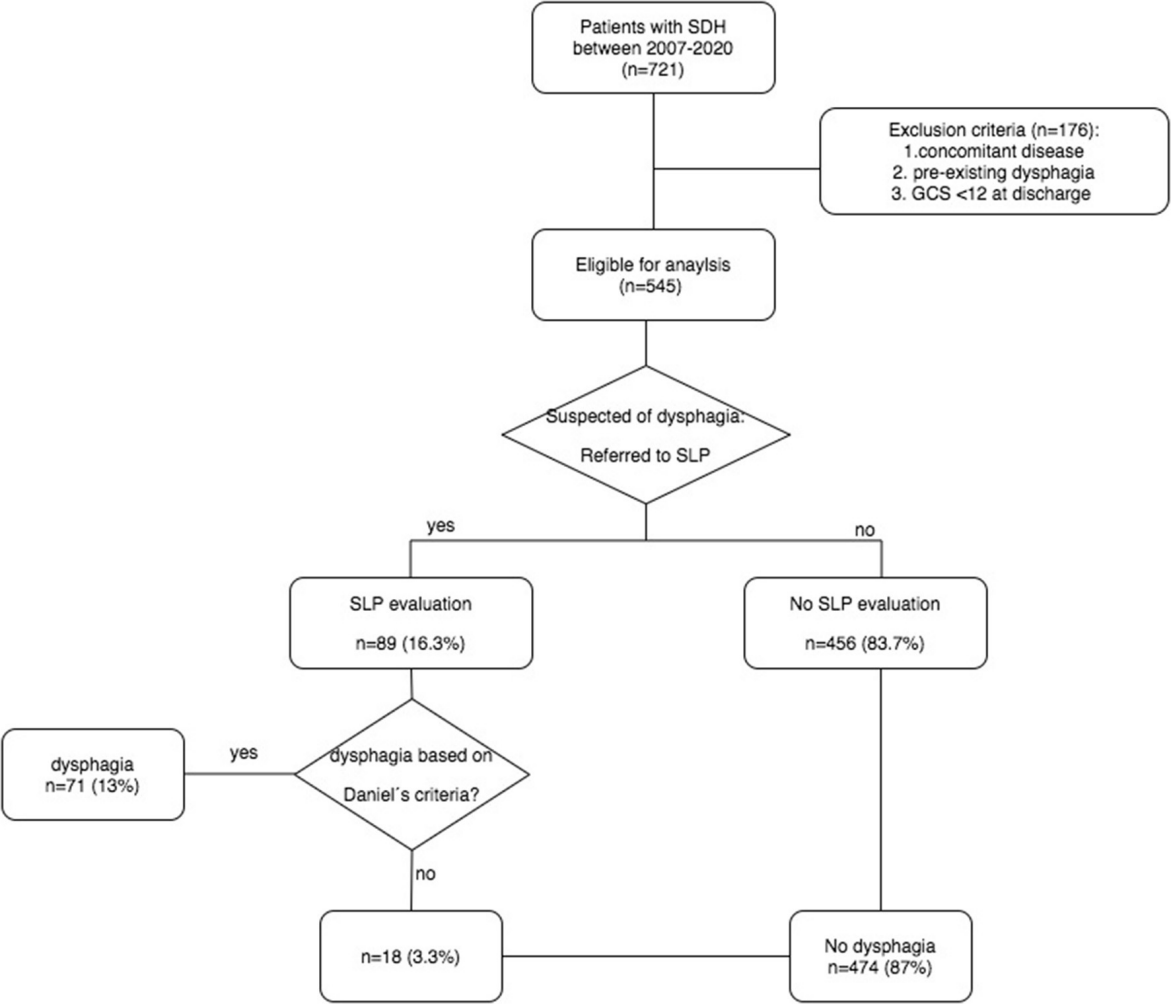
Flow-chart of dysphagia screening. SDH, subdural hematoma; GCS, Glasgow Coma Scale; SLP, Speech-language-pathologist.

All patients admitted to the neurosurgical department of authors' institute between January 2007 and June 2020 with diagnosis of subdural hematoma were retrospectively evaluated with the following inclusion criteria: (1) acute or chronic subdural hematoma diagnosed by CT- or MRI-scan, and (2) patients aged 18 years and above. Patients with pre-existing dysphagia based on the history of the patient, concomitant disease affecting the central nervous system (for example, intracerebral hemorrhage, stroke, and tumor), or postoperative Glasgow coma scale (GCS) <12 were excluded. Figure 1 illustrates the study flow in detail.

Based on electronic database and charts of patients, the following variables were obtained: age, sex, type of SDH, preoperative and postoperative GCS, comorbidities (e.g., hypertension, atrial fibrillation, diabetes mellitus, cardiovascular, respiratory, renal, dementia, metabolic, hematologic, and remote stroke), volume of SDH, midline shift, infection (pneumonia, urinary tract infection, and sepsis), and type of treatment (operation/conservative).

Functional outcome was assessed by Glasgow outcome scale (GOS). The GOS 4–5 was considered a favorable outcome.

### Dysphagia Assessment

All patients with clinical suspicion on dysphagia were referred to an experienced SLP. In a first step, evaluation of the patients' state of consciousness was performed. Patients who were awake and alert for at least 15 min received a standardized 50 ml water swallowing test (WST). Consequently, all patients with GCS <12 postoperatively were excluded as mentioned above. Patients were classified as dysphagic if 2 out of the following 6 items were present: dysarthria, dysphonia, abnormal gag reflex, abnormal cough, cough after swallow, and wet voice (any 2 as described by Daniels et al.) (9). Orofacial muscular symmetry, strength, and sensation as well as cranial nerve function were also assessed.

Patients with dysphagia received treatment by an SLP during their hospitalization. Severity of oral intake was rated according to the swallowing disorder scale (“Schluckbeschwerdenskala” = SBS, Prosiegel et al. 2002) (10). The definition of SBS is described in Supplementary Table 1 in detail.

### Imaging Assessment

The MRI or CT scan was performed based on a standardized in-house protocol. The volume of SDH was measured by using a transverse image of those scans with a simple ABC/formula as previously published (11). Midline shift was measured at the level of the pineal gland.

### Study Design

Depending on the dysphagia assessment, patients were divided into two groups for further analysis: dysphagia and non-dysphagia group. By doing so, the type of treatment was an independent predictor for dysphagia, wherefore; we performed a subgroup analysis by dividing those into operative and conservative arms.

The goal of the study was mainly to evaluate the prevalence of dysphagia in SDH; second, to identify independent predictors for dysphagia; third, to observe the natural course of dysphagia after operation and conservative treatment under SLP treatment, and lastly, to evaluate the outcome in patients with and without dysphagia.

### Statistical Analysis

The IBM SPSS Statistics (Version 22, IBM Corp., Armonk, NY, USA) was used for data analysis. Data were described using median ± interquartile range (IQR) and numbers of patients, including percentages, for categorical variables. Univariate and multivariate logistic regression analyses were performed to obtain independent predictors for the dysphagia. For independent non-parametric parameters, the Mann–Whitney *U*-test was used; and for dependent, non-parametric samples, the Wilcoxon Sign Test, was used. For binary parameters, variables were analyzed in a contingency table using χ^2^ test. To assess the impact of the variables, odds ratios with 95% confidence intervals were calculated. A *p*-value < 0.05 was considered as statistically significant and all tests were 2-tailed.

### Data Availability

Anonymized data will be shared by request from any qualified investigators for purposes of replicating procedures and results.

## Results

Overall, 721 patients with diagnosis of SDH were identified between January 2007 and June 2020. Among them, 176 patients (24.4%) were excluded due to concomitant disease, pre-existing dysphagia, or GCS <12 at discharge. Consequently, 545 patients (75.6%) were eligible for the analysis (Figure 1). Median age was 77 (IQR 68–83) and women to men ratio was 1.8:1. Acute SDH was diagnosed in 139 patients (25.5%) and chronic SDH in 406 patients (74.5%). Out of 545, 476 patients (87.3%) were surgically treated, whereas the remainder (12.7%) received conservative treatment due to small volumes of SDH, or clinically inapparent SDH. Baseline characteristics and clinical variables are displayed in Table 1.

**Table 1:** Baseline characteristics of the study population.

**Variables**	**Total**	**Dysphagia**	**No. dysphagia**	***P* value^+^**
*n* (%)	545	71	474	
Median age, y (IQR) Age >77y	77 (68–83) 269 (49.4)	77 (68–83) 43 (60.6)	77 (68–83) 226 (47.7)	0.270 0.043^*^
Women, *n* (%)	193 (35.4)	47 (66.2)	305 (64.3)	0.761
Comorbidities, *n* (%)				
Hypertonus	361 (66.2)	54 (76.1)	307 (64.8)	0.061
Atrial fibrillation	110 (20.2)	23 (32.4)	87 (18.4)	0.006^*^
Diabetes mellitus typII	107 (19.6)	17 (23.9)	90 (18.9)	0.327
Cardiovascular	140 (25.7)	35 (49.3)	105 (22.2)	<0.001^*^
Respiratory	46 (8.4)	9 (12.7)	37 (7.8)	0.169
Renal	61 (11.2)	12 (16.9)	49 (10.3)	0.102
Dementia	46 (8.4)	4 (5.6)	42 (8.9)	0.362
Metabolic	162 (29.7)	16 (22.5)	146 (30.8)	0.155
Hematologic	49 (9.0)	5 (7.0)	44 (9.3)	0.538
Remote stroke	51 (9.4)	11 (15.5)	40 (8.4)	0.057
GCS at admission, *n* (%)				
3–6	39 (7.2)	13 (18.3)	26 (5.5)	<0.001^*^
7–12	62 (11.4)	13 (18.3)	49 (10.3)	0.048^*^
13–15	444 (81.5)	45 (63.4)	399 (84.2)	<0.001^*^
Subdural hematoma				
Acute, *n* (%)	139 (25.5)	40 (56.3)	99 (20.9)	<0.001^*^
Chronic, *n* (%)	406 (74.5)	31 (43.7)	375 (79.1)	<0.001^*^
Unilateral, *n* (%) Right, *n* (%) Left, *n* (%)	423 (77.6) 119 (21.8) 304 (55.8)	56 (78.9) 28 (39.4) 28 (39.4)	367 (77.4) 91 (19.2) 276 (58.2)	0.785 <0.001 <0.001
Bilateral, *n* (%)	122 (22.4)	15 (21.1)	107 (22.6)	0.785
Volume, cm^3^ (SD)	119.1 ± 65	114.9 ± 70.5	119.7 ± 64.3	0.466
Midline shift, mm (SD)	6.9 ± 4.8	6.3 ± 4.7	7.0 ± 4.8	0.365
Infection, *n* (%)				
Pneumonia	53 (9.7)	20 (28.2)	33 (7.0)	<0.001^*^
Urinary tract infection	36 (6.6)	10 (14.1)	26 (5.5)	0.001^*^
Sepsis	2 (0.4)	0 (0)	2 (0.4)	0.583
Treatment				
Surgery	476 (87.3)	56 (78.9)	420 (88.6)	0.021^*^
Conservative	69 (12.7)	15 (21.1)	54 (11.4)	0.021^*^

+ *Chi-square test was used for binary parameters. Mann-Whitney-U test was used for continuous parameters.*

* *p < 0.05 was defined as statistically significant. N, number; y, years; IQR, interquartile range; GCS, Glasgow Coma Sclae*.

Out of 545, 89 patients (16.3%) had clinical suspicion of dysphagia, wherefore, they were referred to an SLP. Dysphagia could be confirmed in 71 patients, reflecting a prevalence of 13%. Both patients with no clinical suspicion of dysphagia and negative dysphagia assessment were classified to the non-dysphagia group (474 patients: 87%) (Figure 1).

Several variables were associated with dysphagia in the univariate analysis: age >77 y (OR 1.69 Cl 95% 1.01–2.80; *p* = 0.04), atrial fibrillation (OR 2.13 Cl 95% 1.23–3.69; *p* = 0.006), cardiovascular disease (OR 3.4 Cl 95% 2.05–5.71; *p* < 0.001), GCS 3-6 at admission(OR 3.86 Cl 95% 1.88–7.93; *p* < 0.001), GCS 13–15 at admission (OR.33 Cl 95% 0.19–0.56; *p* < 0.001), acute SDH (OR 4.89 Cl 95% 2.91–8.21; *p* < 0.001), chronic SDH (OR.20 Cl 95% 0.12–0.34; *p* < 0.001), pneumonia (OR 5.24 Cl 95% 2.80–9.81; *p* < 0.001), urinary tract infection (OR 2.8 Cl 95% 1.30–6.14; *p* = 0.001), operation (OR.48 Cl 95%0.25–0.91; *p* = 0.001), and conservative treatment (OR 2.08 Cl 95% 1.10–3.94; *p* = 0.021). In a subgroup analysis concerning unilateral SDH, right-sided SDH was an additional predictor for dysphagia compared to left-sided SDH (OR 2.7 CI 95% 1.62–4.65; *p* < 0.001).

In the logistic regression analysis, GCS <13 at admission (OR 4.17 Cl 95% 2.70–6.41; *p* < 0.001), cardiovascular disease (OR 2.29 Cl 95% 1.37–3.85; *p* = 0.002), and pneumonia (OR 2.88 Cl 95% 1.36–3.85; *p* = 0.002) were identified as predictors for dysphagia. Surgical treatment (OR 0.23 Cl 95% 0.15–0.35; *p* < 0.001) was the only protective factor for dysphagia.

**Figure 2:**
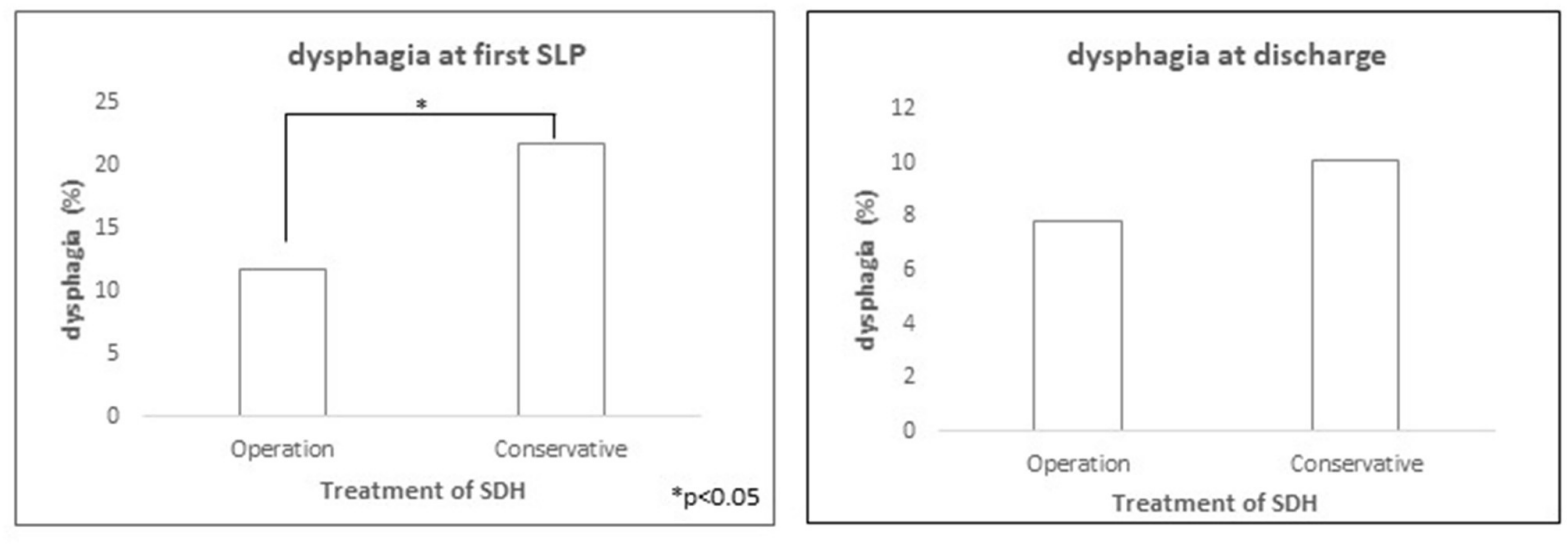
Significantly higher rate of dysphagia in patients with conservative treatment compared to operative treatment of subdural hematoma at first SLP evaluation (*p* < 0.05). At discharge, the rate of dysphagia was comparable for both groups (*p* = n.s.).

**Figure 3:**
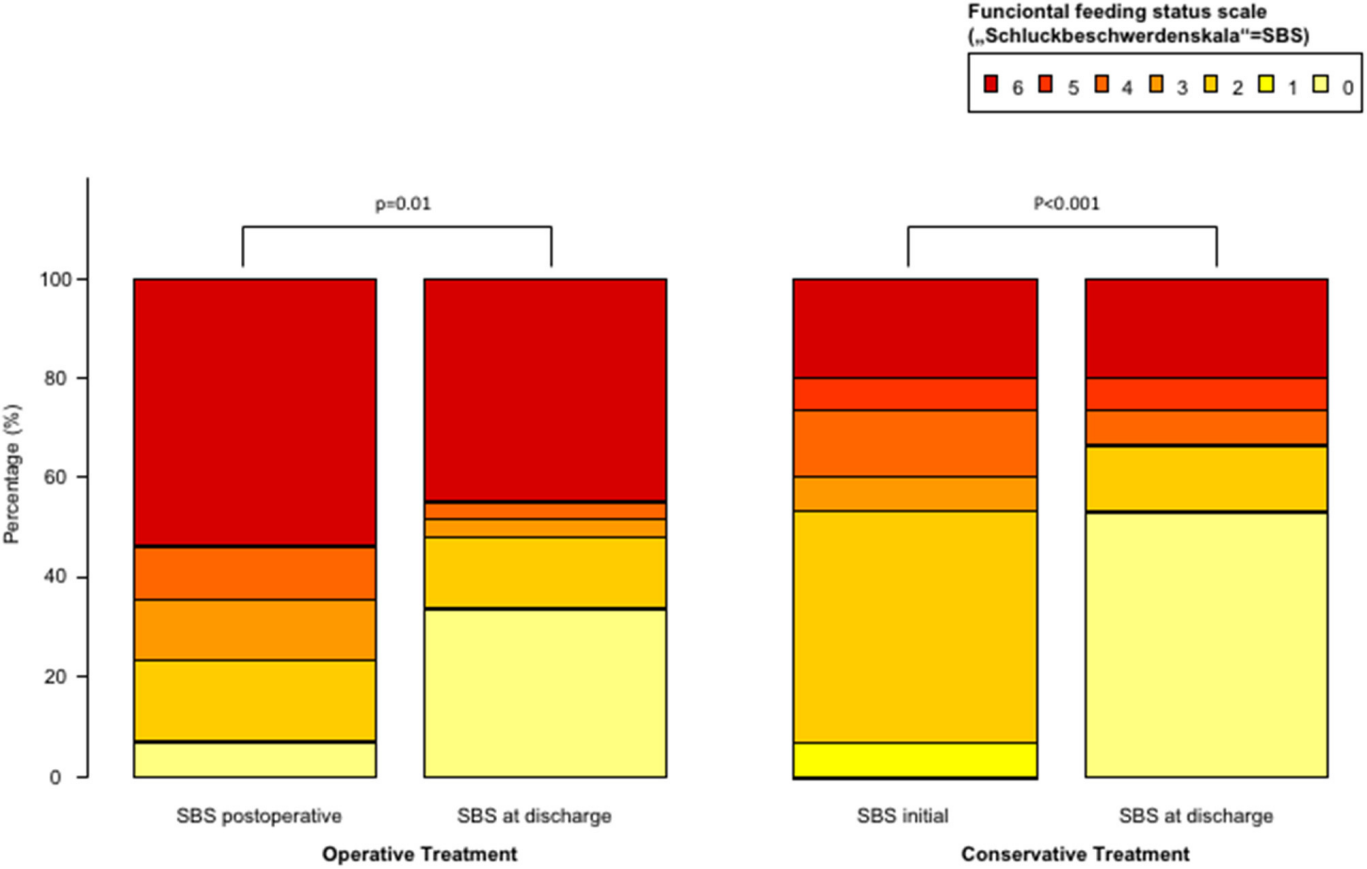
Functional feeding status scale in patients with dysphagia divided by operative and conservative treatment of subdural hematoma.

Since the type of treatment was shown as an independent predictor for dysphagia, we divided patients into an operative and a conservative arm in the subgroup analysis. The incidence of dysphagia was significantly lower in the operative arm compared to the conservative arm (11.8 vs. 21.8%; *p* = 0.02) (Figure 2). Under SLP treatment, dysphagia rate at discharge was comparable in both arms (7.8 vs. 10.1%; *p* > 0.05), which presented an overall significant improvement of the initially diagnosed dysphagia (11.8 to 7.8%; *p* = 0.04; 21.8 to 10.1%; *p* = 0.06). Similarly, functional feeding status scale (SBS) showed significant improvement between initial SLP assessment and at discharge in both arms (Figure 3). However, if dysphagia was present, the majority of patients from the operative arms had SBS 6 representing the most severe dysphagia, which hardly improved at discharge. In contrast, moderate dysphagia, with SBS between 1 and 4, improved significantly at short-term follow-up in both arms.

All in all, favorable outcome (GOS 4–5) was achieved in 430 of 545 patients (78.9%). Patients with dysphagia had significantly worse outcome (34 of 71 patients, 48.8%) compared to patients without dysphagia (81 of 474 patients, 26.4%; *p* < 0.001). None of those patients died at discharge.

## Discussion

This study investigated the frequency of SDH-related dysphagia, as well as its clinical and radiological predictors, in a large cohort of patients. Our data show that dysphagia affects about 13% of patients with SDH. In contrast to previous studies which focused on traumatic brain injury in general, this is, to the best of our knowledge, the first systematic investigation on dysphagia in patients suffering from acute and chronic SDH. On the contrary, the reported high prevalence of swallowing dysfunction in TBI dysphagia frequency is significantly lower in patients with SDH, suggesting that the type of brain injury plays a pivotal role in the development of swallowing impairment in this respective cohort. Nevertheless, dysphagia has a great impact on the outcome as patients with dysphagia are three times more likely to develop pneumonia with those subjects, with a verified aspiration harboring an 11-fold increased risk (3). Moreover, studies have reported a significantly higher morbidity, 5-year mortality, and also higher hospitalization costs in patients with dysphagia-associated pneumonia (12, 13). In this context, early identification of dysphagia in SDH is eminent regarding nutritional management and an absolute risk reduction of pneumonia incidence of 1% per day (14).

Among clinical predictors, the GCS <13 at admission and in cardiovascular disease were significantly associated with dysphagia in SDH. This is in line with previous studies where cardiovascular disease are identified as predisposing risk factor for dysphagia (15). Furthermore, our data show a significant correlation between dysphagia and the severity of head trauma expressed by GCS. It is well known that the diffuse axonal injury, which could involve both periventricular and subcortical regions, is a remarkable complication following a traumatic brain injury causing variable symptoms like behavioral problems, movement disorders, dysarthria, and dysphagia (16). In particular, affection of pyramidal tract disrupting the connecting fibers of swallowing centers in the cortex and brainstem could result in speech and swallowing disorders.

Additionally, our data show a significant correlation between the presence of dysphagia and right hemispheric SDH. This is in line with previous studies conducted in patients who had stroke, identifying multiple lesions within the right hemisphere being associated with a higher rate of dysphagia, and long-lasting and more severe swallowing dysfunction (17).

Although radiological parameters like midline shift and lesion volume showed no significant correlation with swallowing function, dysphagia frequency was significantly lower in patients who received surgical treatment of SDH. This might lead to the assumption that a surgical evacuation, reducing intracranial pressure, has a beneficial impact on swallowing function.

Furthermore, our data show that chronic SDH was a protective factor for dysphagia in contrast to acute SDH. The main reason for this phenomenon could be that an acute disruption of the functional connectivity of the cortical and subcortical swallowing network more likely causes dysphagia that is similar to the development of stroke-related dysphagia. In contrast, chronic SDH develops over a longer period of time, thus, allowing the swallowing network to adjust and compensate (18).

However, up to date, the nature of dysphagia in SDH is not fully understood. Multiple cortical structures such as the opercular-insular region have been identified as critical nodes of the supratentorial deglutition network (19, 20). Additionally, several subcortical regions like basal ganglia, corona radiata, thalamus, and internal capsule are known to play an important role in the deglutition network as well (21). In case of stroke or intracerebral hemorrhage, the damaged location refers to a certain area, whereas in case of SDH, the whole hemisphere is affected by the space-consuming effect of hematoma. This might affect multiple regions of the deglutition network resulting in dysphagia.

In patients with mild to moderate dysphagia, we observed a significant improvement of swallowing function after short-term follow-up. This supports the assumption that the compressive effect of SDH might be the leading cause of dysphagia in contrast to irreversible brain injury. However, patients with severe dysphagia hardly improved regardless of their operative and conservative treatment.

Limitations of this study are its retrospective design and lack of instrumental swallowing assessment, which is known to offer a higher sensitivity in detecting dysphagia and dysphagia severity. “Therefore, our observations should be validated in a prospective study using instrumental swallowing diagnostic (e.g., fiberoptic endoscopic evaluation of swallowing or video fluoroscopy). This would allow for an in-depth characterization of dysphagia features in these patients.”

Moreover, the dysphagia assessment was not performed in all patients, which might lead to a selection bias. Nevertheless, we could observe swallowing impairments in 13% of patients, which is quite a considerable proportion. Incidence of dysphagia might be even higher if SLP assessment would have been conducted in all patients, which should be investigated in following studies.

## Conclusion

Neurogenic dysphagia is a frequent finding in SDH. It is associated with pneumonia and worse functional outcome. Age and GCS at admission might prove useful in clinical decision pathways to risk-stratify identify the patients that need a thorough swallowing assessment by an SLP after surgery. Operative treatment is effective in preventing dysphagia and should be considered in high-risked patients to improve the clinical course and outcome.

## Data Availability Statement

The original contributions presented in the study are included in the article/Supplementary Material, further inquiries can be directed to the corresponding author/s.

## Ethics Statement

The studies involving human participants were reviewed and approved by the local Ethics Committee of Goethe University Frankfurt, Frankfurt am Main, Germany (IRB 20-826). Written informed consent for participation was not required for this study in accordance with the national legislation and the institutional requirements.

## Author Contributions

S-YW: study design, data collection, data analysis, and manuscript writing. SK: data collection and data analysis. DD: data collection and critical revision. FG: critical revision and supervision. BB: data collection and statistical analysis. TF: study concept and critical revision. JK: study design and critical revision. VS: critical revision. SL: study design, data analysis, critical revision, and supervision. All authors contributed to the article and approved the submitted version.

## Conflict of Interest

The authors declare that the research was conducted in the absence of any commercial or financial relationships that could be construed as a potential conflict of interest.

## Publisher's Note

All claims expressed in this article are solely those of the authors and do not necessarily represent those of their affiliated organizations, or those of the publisher, the editors and the reviewers. Any product that may be evaluated in this article, or claim that may be made by its manufacturer, is not guaranteed or endorsed by the publisher.
