# Supplementary material for: Neurogenic Dysphagia in Subdural Hematoma
Source: Front Neurol. 2022 Jan 26;12:701378. doi: 10.3389/fneur.2021.701378 (PMC8826688; doi:10.3389/fneur.2021.701378)
Supplement: Supplementary file 1 [file Table_1.docx]

**Supplementary Table 1**. Functional feeding status scale („Schluckbeschwerdenskala“=SBS) (10)

| **SBS value** | **Functional feeding status** |
| --- | --- |
| 0 | Complete oral feeding without limitatoin |
| 1 | Complete oral feeding with compensation* without limitation of food consistency |
| 2 | Complete oral feeding without compensation with limitation of food consistency |
| 3 | Complete oral feeding with compensation and limitation of food consistency |
| 4 | Partial oral feeding |
| 5 | Partial oral feeding with compensation |
| 6 | Feeding only by gastric tube |

*compensation means postural change or certain swallowing technique
